# A bacterial metabolite induces glutathione-tractable proteostatic damage, proteasomal disturbances, and PINK1-dependent autophagy in *C. elegans*

**DOI:** 10.1038/cddis.2015.270

**Published:** 2015-10-15

**Authors:** B A Martinez, H Kim, A Ray, G A Caldwell, K A Caldwell

**Affiliations:** 1 Department of Biological Sciences, The University of Alabama, Tuscaloosa, AL, USA; 2 Departments of Neurobiology and Neurology, Center for Neurodegeneration and Experimental Therapeutics, University of Alabama at Birmingham, Birmingham, AL, USA

## Abstract

Gene-by-environment interactions are thought to underlie the majority of idiopathic cases of neurodegenerative disease. Recently, we reported that an environmental metabolite extracted from *Streptomyces venezuelae* increases ROS and damages mitochondria, leading to eventual neurodegeneration of *C. elegans* dopaminergic neurons. Here we link those data to idiopathic disease models that predict loss of protein handling as a component of disorder progression. We demonstrate that the bacterial metabolite leads to proteostatic disruption in multiple protein-misfolding models and has the potential to synergistically enhance the toxicity of aggregate-prone proteins. Genetically, this metabolite is epistatically regulated by loss-of-function to *pink-1*, the *C. elegans* PARK6 homolog responsible for mitochondrial maintenance and autophagy in other animal systems. In addition, the metabolite works through a genetic pathway analogous to loss-of-function in the ubiquitin proteasome system (UPS), which we find is also epistatically regulated by loss of PINK-1 homeostasis. To determine remitting counter agents, we investigated several established antioxidants and found that glutathione (GSH) can significantly protect against metabolite-induced proteostasis disruption. In addition, GSH protects against the toxicity of MG132 and can compensate for the combined loss of both *pink-1* and the E3 ligase *pdr-1*, a Parkin homolog. In assessing the impact of this metabolite on mitochondrial maintenance, we observe that it causes fragmentation of mitochondria that is attenuated by GSH and an initial surge in PINK-1-dependent autophagy. These studies mechanistically advance our understanding of a putative environmental contributor to neurodegeneration and factors influencing *in vivo* neurotoxicity.

Protein homeostasis (proteostasis) encompasses the process of translation, folding, compartmentalization, and degradation of proteins to maintain the long-term survival and functionality of the cell.^1, 2, 3^ When proteins become misfolded they must be refolded or degraded to prevent disruptions to critical processes that result from proteotoxic stress.^3, 4^ Surveillance machinery that combats proteotoxic stress includes the ubiquitin proteasome system (UPS), retrograde chaperone-inducing signaling systems termed unfolded protein responses (UPR), and bulk destruction through autophagy. The cell also utilizes protein clearance machinery to induce the destruction of entire organelles, such as mitochondria, when they no longer function correctly^5, 6^ to protect the cell from reactive oxygen species (ROS). The last line of defense includes antioxidants in order to maintain a reduced intracellular state and attenuate damage to proteins.^7, 8, 9, 10, 11^ Often, these regulated mechanisms are challenged by both the environment and genetic susceptibility factors. The integration of both, via gene-by-environment interactions, has been hypothesized to underlie many idiopathic neurodegenerative disorders.^12, 13, 14^ Understanding how the environment contributes to disease pathologies is important for understanding neurodegeneration.

Sources of environmental stressors are understudied and largely limited to human-derived toxicants such as pesticides like rotenone.^14, 15^ However, people living in agricultural environs are often at a greater risk of developing neurodegenerative disorders that cannot be accounted for by human-derived toxicants alone.^16^ Environmental contributors may come from natural sources like metabolite-producing bacteria. For instance, bacterial sources have been reported to induce DOPA-responsive movement disorders in mice.^17^ Mechanistically, competition strategies among bacteria that produce antibiotics and small metabolites like phenazines that limit the growth of other bacterial species may have off-target effects on mitochondrial homeostasis, leading to ROS, protein damage, and neurodegeneration.^18^ Indeed, proteostatic dysfunction, altered mitochondrial dynamics, and elevated ROS production are characteristics of sporadic Parkinson's disease (PD).^19, 20, 21^

Our laboratory previously demonstrated neurodegeneration induced by unreported small compounds within the growth media of the Gram-positive soil bacterium *Streptomyces venezuelae*.^22, 23, 24^ These bacterial products induce neuronal death in both *C. elegans* and cultured human neurons,^22^ disrupt mitochondrial complex I, induce ROS, and decrease ATP production.^25^ However, how these observations link to protein homeostasis has not been explored. Here we report that the active fraction of the *S. venezuelae* media induces disruptions in protein homeostasis, glutathione (GSH)-tractable *α*-synuclein toxicity, that UPS disruptions are epistatically regulated by loss-of-function to the PARK9 homolog *pink-1*, and that PINK1-dependent autophagy results in mitochondrial morphology disruptions. These observations indicate that *pink-1* and UPS functionality are required for metabolite-induced protein toxicity in *C. elegans*, suggesting that these pathways may be linked and that environmental contributors to neurodegenerative disease may proceed through pathways implicated in familial forms.

## Results

### The *S. venezuelae* metabolite synergistically enhances toxicity associated with pathogenic protein expression in *C. elegans* neurons

*S. venezuelae* active fraction containing a small secondary product (MW<300) is isolated following growth of cells in liquid culture through extraction using dichloromethane (DCM) to separate compounds from the aqueous phase. The DCM fraction is evaporated completely and the solidified substance is resuspended in ethyl acetate (EtAc) as described;^25^ hereafter (for brevity and consistency) it will be referred to as the metabolite. EtAc is used as a negative solvent control in experiments and does not cause significant neurodegeneration.^25^

**Figure 1:**
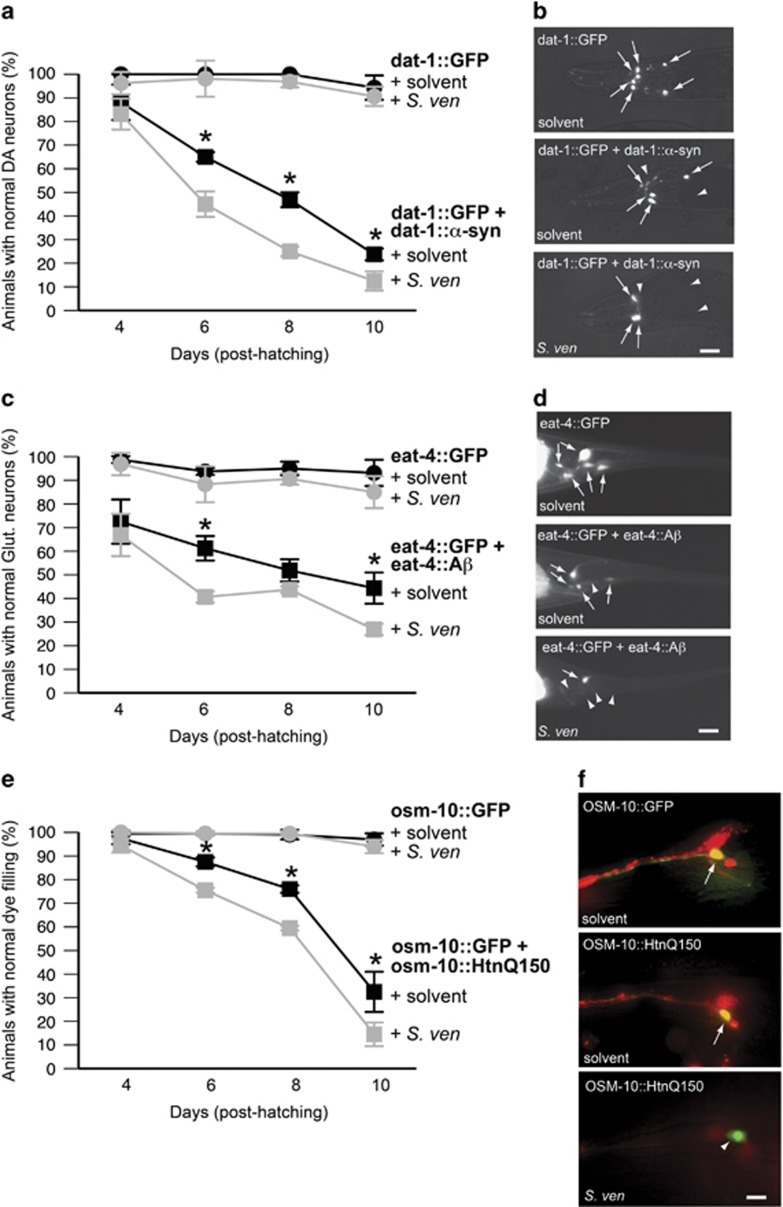
The *S. venezuelae* metabolite synergistically enhances pathogenic protein toxicity in *C. elegans* neurons. Animals were treated chronically with 100 *μ*l metabolite/EtAc (*S. ven*) solution [5 *μ*l of the 1000 × concentrated metabolite/ml] or EtAc alone (solvent) on 35 mM plates as described in Supplementary Figure S1b. This solution is added to the grown OP50 bacterial lawn before animal transfer and the solvent is dried before use. (**a**) Animals expressing GFP alone or *α*-synuclein (*α*-syn) and GFP using the P_*dat-1*_ promoter to target expression to dopaminergic neurons were assayed for altered neurodegeneration in response to *S. ven* at days 4, 6, 8, and 10 post hatching. (**b**) Animals without *α*-syn always display six anterior dopaminergic neurons (arrows), however neuron cell death is induced by *α*-syn expression (arrow heads) in a time- and metabolite-dependent manner. Scale bar, 10 *μ*m. (**c**) Animals expressing GFP alone or the human A*β*_42_ peptide using the P_*eat-4*_ promoter to target expression to glutamatergic neurons were assayed for altered neurodegeneration in response to *S. ven* at days 4, 6, 8, and 10 post hatching. (**d**) Animals without A*β*_42_ always display five posterior glutamatergic neurons (arrows), however neuron cell death is induced by A*β*_42_ expression (arrowheads) in a time- and *S. ven-*dependent manner. Scale bar, 10 *μ*m. (**e**) Animals expressing GFP alone or mutant huntingtin (HtnQ_150_) and GFP using the P_*osm-10*_ promoter to drive expression to the ASH-sensory neuron were assayed for altered dye-filling behavior in response to *S. ven* at days 4, 6, 8, and 10 post hatching. (**f**) Animals without HtnQ_150_ display co-localization (arrow) in the ASH neuron of the endogenous GFP with the red DiI lipophilic dye (100 ng/*μ*l), which inundates sensory neurons with exposed ciliary endings such as ASH, however loss of dye-filling (arrow head) as a result of neuron damage is induced by HtnQ_150_ in a time- and *S. ven-*dependent manner. Scale bar, 20 *μ*m. Quantitative data in the above panels are represented as the mean±S.D.; *n*=40 animals per treatment per strain, replicated 3–4 times and analyzed using two-way ANOVA with Tukey's *post hoc* test, *P<0.05

PD is characterized by dopaminergic neuron loss^14^ and is associated with *α*-synuclein, which induces neurodegeneration when overexpressed or mutated.^26^ Consistent with this, *α*-synuclein overexpression in worm dopaminergic neurons using the *dat-1* promoter (P_*dat-1*_) induces neurodegeneration in the six anterior dopaminergic neurons (Supplementary Figure S1a).^2, 27^ We performed a timecourse experiment in this genetic background and found that chronic metabolite supplementation (Supplementary Figure S1b) significantly decreased the percentage of animals with normal dopaminergic neurons at days 6–8 post hatching (Figures 1a and b). Animals at day 4 do not display enhanced neurodegeneration, suggesting that *α*-synuclein accumulation might not be sufficient for neurotoxicity manifestation. Animals expressing only GFP do not exhibit neurodegeneration at the time points and concentrations examined, potentially indicating a synergistic interaction between *α*-synuclein and the metabolite.

To determine whether these effects were limited to *α*-synuclein-induced neurodegeneration, we performed timecourse exposures in two other neurodegeneration models. First, human amyloid-*β* peptide (A*β*_42_), a toxic cleavage product of the amyloid precursor protein associated with Alzheimer's disease^3, 28^ was examined in five glutamatergic tail neurons using the *eat-4* promoter (P_*eat-4*_) (Supplementary Figure S1a). This expression induces neurodegeneration in a time-dependent manner and is modulated by factors, which promote proper proteostasis.^29^ Transgenic animals with A*β*_42_ expression had significantly decreased glutamatergic neuron counts at days 6 and day 10 when exposed to metabolite (Figures 1c and d). The metabolite had no effect on glutamatergic health in the absence of A*β*_42_. Second, we examined metabolite effects on mutant huntingtin (Htn-Q_150_) expression under the control of the *osm-10* promoter (P_*osm-10*_) in the *C. elegans* ASH-type sensory neuron.^30^ We assayed animals for defects in lipophilic dye uptake reported to be associated with Htn-Q_150_-induced disruption of ciliary endings. We discovered that dye-filling defects were significantly greater in metabolite-treated animals at days 6–10 (Figures 1e and f). Animals without pathogenic proteins do not display neurodegeneration. Taken together, these data suggest that the metabolite can enhance toxicity of neuronally-expressed pathogenic proteins *in vivo*.

### The *S. venezuelae* metabolite is associated with proteostasis disruption

**Figure 2:**
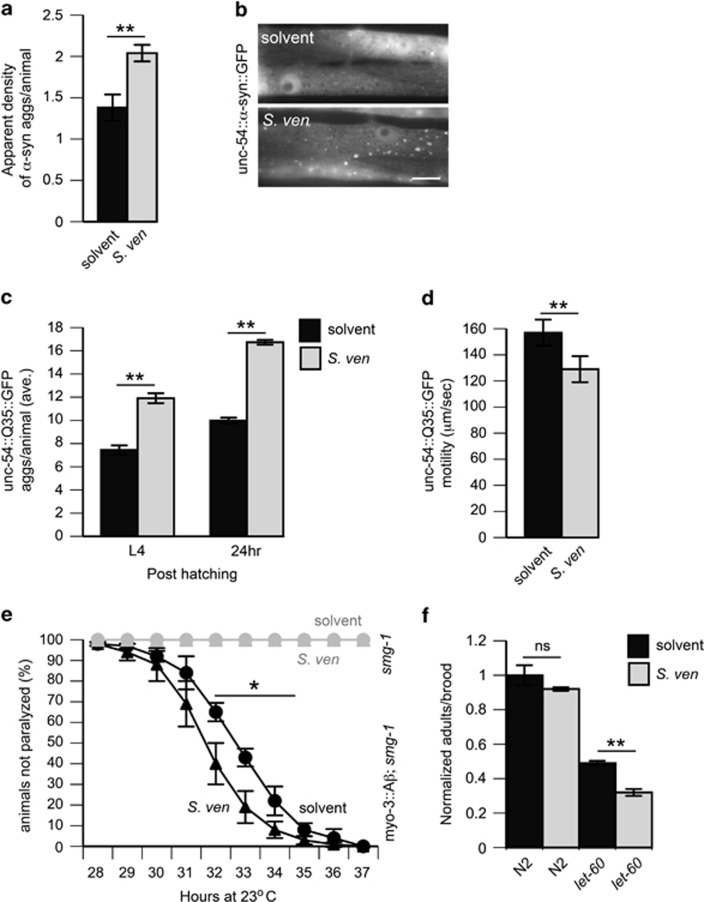
The *S. venezuelae* metabolite induces proteostasis disruption. Observation of pathogenic protein aggregation is best studied in young animals (~day 3 post hatching) as older animals tend to have saturated aggregation. Therefore, it was necessary to expose animals to a higher metabolite concentration in a dosing regimen referred to as ‘semi-acute' (Supplementary Figure S1c) for examination of metabolite effects in this context. Animals for these assays were treated in a semi-acute manner by soaking L1-synchronized animals in metabolite solution [10–15 *μ*l of the 1000 × concentrated metabolite/ml] or unextracted conditioned *S. ven* media (only for the A*β* paralysis experiment displayed in e) for 8 h. Solvent treatment for A*β* paralysis was SYZ media in artificial seawater used to grow the *S. ven* bacteria, otherwise solvent treatment refers to EtAc/buffer. Animals were assayed after semi-acute treatment at various time points for proteostasis disruption signified by loss of protein degradation and handling. (**a**) Animals expressing *α*-syn conjugated to GFP in the *C. elegans* body muscle cells under the control of the P_*unc-54*_ promoter were assayed 2.5 days (60 h) after treatment for apparent aggregate density using a qualitative 0–3 scale with the experimenter blind to the treatment condition being analyzed. Data represented as mean±S.E.M.; *n*=30 animals per treatment assessed in 3–4 replicates. **P<0.01 Student's *t*-test. (**b**) Representative *C. elegans* bodywall muscle cells expressing *α*-syn::GFP treated with solvent or *S. ven* metabolite. Scale bar, 5 *μ*m. (**c**) Animals expressing a polyglutamine-35 tract (Q_35_) conjugated to YFP within the *C. elegans* body muscle cells under the control of the P_*unc-54*_ promoter were treated with solvent or metabolite. They were assayed at time points after treatment corresponding to the L4 larval stage and 24 h after L4 by counting the number of aggregates present per animal. Data represented as mean±S.E.M.; *n*=30 animals per treatment assessed in 3–4 replicates. **P<0.01. Data were assessed by Student's *t*-test. (**d**) Animals bearing PolyQ_35_ in the bodywall muscle cells were exposed to the metabolite or solvent control and then examined with the MBF Bioscience Wormlab System for motility (*μ*M/second) on a clean agar plate. *n*=40–50 animals. Data represented as mean±S.E.M were assessed using Student's *t-*test. (**e**) Animals expressing A*β*_42_ peptide under the control of the bodywall muscle promoter (P_*myo-3*_) using a temperature-repression system (*smg-1*) were upshifted to 23 °C at the L3 larval stage to induce expression of A*β*; treatment is with *S. ven* metabolite or solvent. In parallel, animals that do not express A*β* were treated identically (overlapping gray lines) but did not exhibit paralysis. *n*=90–120 animals per treatment, replicated 3–4 times. Data represented as mean±S.E.M. were assessed using two-way ANOVA and Tukey's *post hoc* test to assess for significance between each time point in the analysis to every other time point. *P<0.05 for time points indicated. (**f**) *let-60(ga89)* brood fecundity was assessed per condition by counting the number of animals within the brood of a single animal that reached adulthood. *n*=24 animal broods per replicate normalized to N2 solvent control. At least three replicates were utilized. Data represented as mean±S.E.M (two-way ANOVA and Tukey's *post hoc* test; **P<0.01)

To determine whether neurodegeneration is correlated with alterations in protein handling, we monitored changes in apparent aggregate density or aggregate count of pathogenic proteins conjugated to fluorescent molecules in muscle cells with a semi-acute regimen of metabolite exposure (Supplementary Figure S1c) and potential behavioral alterations due to protein misfolding. First, we observed that the apparent aggregate density of *α*-synuclein^27^ was significantly increased upon metabolite treatment (Figures 2a and b). Second, animals with fluorescently-conjugated polyQ_35_ and polyQ_40_ had enhanced aggregate formation at two timepoints (Figure 2c; Supplementary Figures S2a and b). Furthermore, metabolite exposure to polyQ_35_ worms significantly impaired motility at the young adult stage (Figure 2d), potentially indicating behavioral alterations in response to aggregation. We did not observe metabolite-induced aggregate formation in subthreshold PolyQ_19_ or GFP only strains (data not shown), indicating that the metabolite alters the ability of threshold-state animals to handle polyQ misfolding. Finally, we extended studies to animals expressing A*β*_42_ in muscle cells under a temperature-sensitive promoter repression system^31^ and found that metabolite exposure induces enhanced paralysis at restrictive temperatures (23–25 °C) but not in animals that do not express A*β*_42_ (Figure 2e; Supplementary Figures S2c and d).

To exclude the possibility of transgenic expression artifacts, we monitored mutant phenotypes of animals bearing metastable protein alleles in RAS, *let-60(ga89),* and paramyosin, *unc-15(e1401*), which are highly sensitive to changes in the protein folding environment.^1^ We found that metabolite exposure in *let-60(ga89)* animals but not in N2 animals significantly decreased brood viability (Figure 2f) and that motility (*μ*m/s) was significantly reduced in *unc-15(e1402)* but not in N2 animals (Supplementary Figure S2e). These observations indicate that broadly applicable proteostasis impairments are a consequence of metabolite exposure.

### GSH attenuates metabolite-associated *α*-synuclein-induced proteotoxicity and proteasomal dysfunction

**Figure 3:**
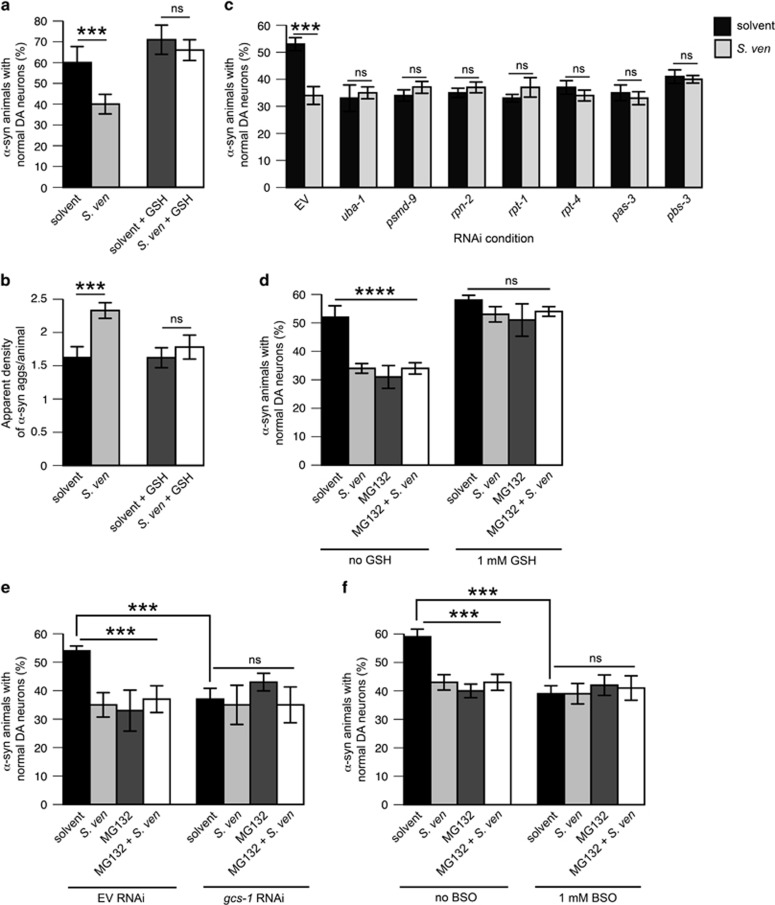
GSH attenuates enhanced *α*-synuclein proteotoxicity and proteasomal dysfunction associated with the metabolite. Nematodes were exposed to the bacterial metabolite chronically for all neurodegeneration assays as described in the Figure 1 legend whereas animals were exposed to the metabolite semi-acutely when they expressed alpha synuclein in bodywall muscle cells, as described in the Figure 2 legend. RNAi was performed in a worm strain whereby RNAi knockdown would occur only in dopaminergic neurons (cell-autonomous RNAi).^33^ (**a**) Animals expressing *α*-syn in the dopaminergic neurons were assessed for neurodegeneration in the context of 1 mM GSH. Data represented as mean±S.D.; *n*=30 animals analyzed per treatment in 3–4 replicates. ***P<0.001 was assessed by two-way ANOVA with Tukey's *post hoc* test. (**b**) Animals expressing *α*-syn in the bodywall muscle cells were assessed for apparent aggregate density in the context of 1 mM GSH. Data represented as mean±S.D.; *n*=30 animals analyzed per treatment in 3–4 replicates. ***P<0.001 was assessed by two-way ANOVA with Tukey's *post hoc* test. (**c**) Animals bearing dopaminergic overexpression of SID-1 (a dsRNA transporter) in a mutant background for *sid-1,* used to selectively target RNAi to dopaminergic neurons in an *α*-syn background were exposed to 1 mM IPTG plates and either empty vector control (EV) or RNAi treatment paradigms affecting the UPS at 6 days post hatching. RNAi was initiated at the L4 larval stage to exclude potential developmental defects. Data represented as mean±S.D.; *n*=30 animals analyzed per treatment in 3–4 replicates. ***P<0.001 was assessed by two-way ANOVA with Tukey's *post hoc* test. (**d**) Animals were treated with 10 *μ*M MG132 with 1 mM GSH in the context of neurodegeneration through enhanced *α*-syn toxicity elicited by the *S. ven* metabolite. EtAc and 0.1% DMSO were used where appropriate to serve as solvent controls. Animals were placed on MG132 concentrations at the larval L4 stage to exclude the possibility of developmental defects. Data represented as mean±S.D.; *n*=30 animals analyzed per treatment in 3–4 replicates. ****P<0.0001 was assessed by two-way ANOVA with Tukey's *post hoc* test. (**e**) Animals were treated with 10 *μ*M MG132 (using 0.1% DMSO as a solvent control) and metabolite as described in (**a**) in the context of cell-autonomous RNAi reduction of the GSH synthesis enzyme *gcs-1* or empty vector control (EV). Data represented as mean±S.D.; *n*=30 animals analyzed per treatment in 3–4 replicates. ***P<0.001 was assessed by two-way ANOVA with Tukey's *post hoc* test. (**f**) Animals were treated with 10 *μ*M MG132 as described in (**a**) in conjunction with 1 mM buthionine sulfoximine (BSO) in the context of neurodegeneration through enhanced *α*-syn toxicity elicited by the *S. ven* metabolite. EtAc and 0.1% DMSO were used where appropriate to serve as solvent controls. Data represented as mean±S.D; *n*=30 animals analyzed per treatment in 3–4 replicates. ***P<0.001 was assessed by two-way ANOVA with Tukey's *post hoc* test

We previously demonstrated that the metabolite increases ROS in *C. elegans* lysates.^25^ To determine whether oxidative damage may be a component of protein mishandling we treated animals to antioxidants and then measured dopaminergic neurodegeneration or *α*-synuclein accumulation. Three antioxidants: ascorbic acid,^9^ uric acid,^8^ and probucol^7, 18^ (Supplementary Figures S3a, c, and d) did not attenuate neurotoxicity whereas melatonin^10^ and GSH^20^ attenuated neurotoxicity (Supplementary Figure S3b,Figure 3a). Only GSH supplementation suppressed enhanced aggregate formation in *C. elegans* bodywall muscle cells (Figure 3b; Supplementary Figures S3e and f).

GSH protects enzymes with open cysteine residues^32^ from oxidative damage, including enzymes of the UPS. In addition, we have previously demonstrated that proteasome impairments may occur from metabolite exposure.^22^ Therefore, to determine whether the metabolite induces proteotoxicity in part through UPS inhibition, we reduced proteasome function with a range of MG132 concentrations and identified that (at concentrations above 5 *μ*M) MG132 induces neurodegeneration in the presence of *α*-synuclein and that this neurotoxicity epistatically regulates metabolite-induced neurotoxicity (Supplementary Figure S4a). To corroborate these findings, we utilized an RNAi strain wherein the effects can be localized specifically to dopaminergic neurons expressing *α*-synuclein^33^ and reduced the sole E1 activating enzyme *uba-1* and six diverse 26S proteasome regulatory subunits (*psmd-9, rpn-2, rpt-1*, *rpt-4*, *pas-3,* and *pbs-3*) in this strain. These gene knockdowns caused enhanced neurodegeneration (Figure 3c). Notably, metabolite activity is attenuated in these backgrounds, indicating that UPS loss-of-function epistatically regulates metabolite activity and suggests that UPS-linked protein homeostasis defects may result from metabolite exposure.

To link proteasomal dysfunction to GSH homeostasis, we treated animals bearing *α*-synuclein with a combination of MG132, metabolite, and GSH and found that exogenous GSH was sufficient to protect against MG132 and/or metabolite toxicity (Figure 3d). In addition, GSH was observed to diminish MG132 and metabolite-induced heightened fluorescence of a proteasome-targeted fluorescence molecule, CFP::CL-1 (degron)^22^ expressed within *C. elegans* dopaminergic neurons (Supplementary Figures S4b and c). Because metabolite toxicity was attenuated by exogenous GSH, we hypothesized that this toxicity may proceed through diminished GSH levels.^34^ To test this, we combined GSH homeostasis impairments with proteasomal disturbances and metabolite exposure in worm neurons expressing *α*-synuclein. In both RNAi of the GSH synthesis gene *gcs-1* in *C. elegans* neurons and exposure to the GSH synthesis inhibitor buthionine sulfoximine (BSO)^35^ we found that combinations with MG132 and/or metabolite operate similarly in their neurodegeneration effect (Figures 3e and f). Furthermore, BSO can decrease proteasomal turnover of CFP::CL-1 in a manner similar to MG132 and metabolite (Supplementary Figure S4d). Therefore, it is possible that GSH homeostasis is a regulator of metabolite-induced proteotoxicity.

### Enhanced *α*-synuclein toxicity is epistatically regulated by the PARK6 homolog, *pink-1*

**Figure 4:**
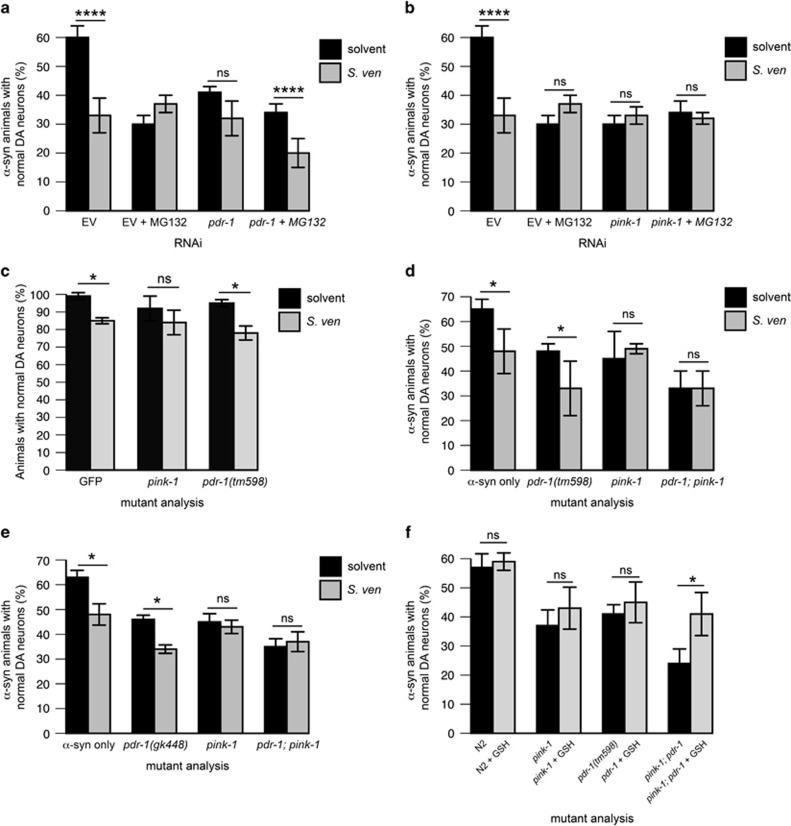
Enhanced *α*-synuclein toxicity is epistatically regulated by the PARK6 homolog, *pink-1.* Neurodegeneration was assessed on animals treated chronically with the bacterial metabolite as described in Figure 1a. RNAi was performed in a strain whereby RNAi knockdown would occur only in dopaminergic neurons (cell-autonomous RNAi).^22^ (**a** and **b**) Animals were reduced through cell-autonomous RNAi for *pdr-1* (RNAi) or *pink-1* (RNAi) in conjunction with the *S. ven* metabolite and/or 10 *μ*M MG132. Animals were treated until day 6 post hatching. Solvent controls include EtAc or 0.1% DMSO. Data represented as mean±S.D.; *n*=30 animals per treatment analyzed in 3–4 replicates. ****P<0.0001 was assessed by two-way ANOVA with Tukey's *post hoc* test. (**c**) Animals with a GFP-only expression construct in dopaminergic neurons were crossed to alleles for *pink-1 (tm1779)* and *pdr-1 (tm598)* mutant lines. These animals were treated with the metabolite until day 12 post hatching. *n*=30 animals analyzed per treatment in 3–4 replicates. *P<0.05 was assessed by two-way ANOVA with Sidak's *post hoc* test. (**d**) Animals with the *pink-1(tm1779)* null mutation and/or the *pdr-1*(*tm598)* deletion were crossed into nematodes expressing both the GFP and *α*-syn transgenes in the dopaminergic neurons. Homozygosity was confirmed by PCR. These animals were treated until day 6 post hatching. Data represented as mean±S.D.; *n*=30 animals analyzed per treatment in 3–4 replicates. *P<0.05 was assessed using two-way ANOVA with Sidak's *post hoc* test. A separate statistical test using two-way ANOVA with Tukey's *post hoc* test (not shown here) demonstrates a statistical difference between solvent-treated *pdr-1* and *pdr-1;pink-1* double-mutation, suggesting an additive phenotype. (**e**) Animals with the *pink-1(tm1779)* null mutation and/or the *pdr-1*(*gk448)* deletion were crossed into nematodes expressing both the GFP and *α*-syn transgenes in the dopaminergic neurons. Homozygosity was confirmed by PCR. These animals were treated until day 6 post hatching. Data represented as mean±S.D.; *n*=30 animals analyzed per treatment in 3–4 replicates. *P<0.05 was assessed using two-way ANOVA with Sidak's *post hoc* test. A separate statistical test using two-way ANOVA with Tukey's *post hoc* test (not shown here) demonstrates a statistical difference between solvent-treated *pdr-1* and *pdr-1;pink-1* double-mutation, suggesting an additive phenotype. (**f**) Strains discussed in (**d**) were treated with 1 mM GSH. Data are represented as mean±S.D.; *n*=30 animals analyzed per treatment in 3–4 replicates. *P<0.05 was assessed using two-way ANOVA with Sidak's *post hoc* test

Given that altered proteasome, protein mishandling, and GSH deficiencies associate with mitochondrial dysfunction in sporadic PD,^11, 20, 21, 32, 36^ we sought to understand how metabolite-induced proteasome inhibition relates to PINK1 and Parkin. These two proteins associate with PD pathogenesis and regulate mitochondrial homeostasis, protein homeostasis, and autophagy in *C. elegans* and other systems.^6, 37, 38, 39, 40, 41^ First, to determine whether metabolite susceptibility depends on UPS-dependent Parkin, we depleted *pdr-1* (the *C. elegans* homolog) cell-autonomously in *α*-synuclein-expressing dopaminergic neurons in conjunction with the metabolite and/or MG132. Knockdown of *pdr-1* plus the addition of all three stressors produced a more severe degeneration phenotype than any two stressors alone (Figure 4a) whereas, with similar conditions, further enhancement of neurodegeneration was not observed in *pink-1*(RNAi) animals (Figure 4b); here, these stressors appeared to act in a related manner. These data indicate that *pink-1*, but not *pdr-1*, may epistatically regulate proteasome inhibition and metabolite-induced protein toxicity.

Although qPCR data indicate that RNAi reduces gene expression by over 80% in *pdr-1* and *pink-1* (RNAi) conditions (Supplementary Figure S5) seemingly parallel regulation of neurodegeneration observed in *pdr-1* (RNAi), and UPS-metabolite toxicity conditions may be due to hypomorphic addition of stressors. Therefore, we crossed our *α*-synuclein-expressing strain to *pink-1(tm1779),* a null allele, and two alleles of *pdr-1: pdr-1(tm598),* an in-frame deletion^40^ and *pdr-1(gk448),* a deletion predicted to remove the start codon for all predicted isoforms. The presence of *α*-synuclein was largely required to parse genetic interactions, as few observable interactions were produced without *α*-synuclein expression in these mutant backgrounds (Figure 4c). Double mutants [*pink-1(tm1779); pdr-1(tm598)* or *pink-1(tm1779); pdr-1(gk448)*] demonstrate significantly greater (*P*<0.05) neurodegeneration than *pdr-1(tm598)* or *pdr-1(gk448)* alone (Figure 4d and e). When considering the interaction between loss of *pink-1* and metabolite addition, these mutant lines confirm RNAi results, suggesting epistatic regulation of metabolite activity by loss of *pink-1* (Figures 4d and e) but not *pdr-1,* which appears to acts in parallel to metabolite toxicity.

At least two possibilities may account for these observations. One possibility is that *pink-1* and *pdr-1* operate independently to regulate *α*-synuclein toxicity in worm dopaminergic neurons and loss of each gene is responsible for an additive phenotype observed in double mutants. Another possibility is that loss of one gene hypersensitizes worm DA neurons to loss of the other, meaning loss of both gene products results in a synergistically toxic state. Within this model, metabolite activity or loss of *pink-1* may be specifically amplified by *pdr-1* loss. To investigate these possibilities, we explored how GSH might protect against *pink-1* and *pdr-1* mutations (Figure 4f). We find that, although GSH cannot protect against *pink-1* or *pdr-1* loss-of-function in the context of *α*-synuclein alone, the joint additive neurodegenerative phenotype of both *pink-1* and *pdr-1* was partially rescued by GSH (Figure 4f) to a degenerative state reminiscent of loss of *pink-1* or *pdr-1* alone. This evidence suggests that the enhanced degeneration observed from loss of *pink-1* and *pdr-1* gene products may not simply be additive but rather arise from a synergistic toxic mechanism that is attenuated by GSH supplementation.

### The metabolite induces mitochondrial morphological dysfunction and PINK-1-dependent autophagy

GSH-tractable UPS inhibition is one mechanism by which the metabolite might exert a toxic influence on *α*-synuclein-induced neurodegeneration. However, proteostasis also involves efficient clearance of defective organellar protein compartments such as the mitochondria^6, 41^ through autophagy. The link between UPS disturbances and autophagic induction remains unresolved, but at least one report has demonstrated that autophagy is induced as a compensatory mechanism for loss of UPS functionality.^42^ To test for altered autophagy, especially of mitochondria, a previously determined metabolite target,^25^ we utilized a combination of molecular and phenotypic assays.

**Figure 5:**
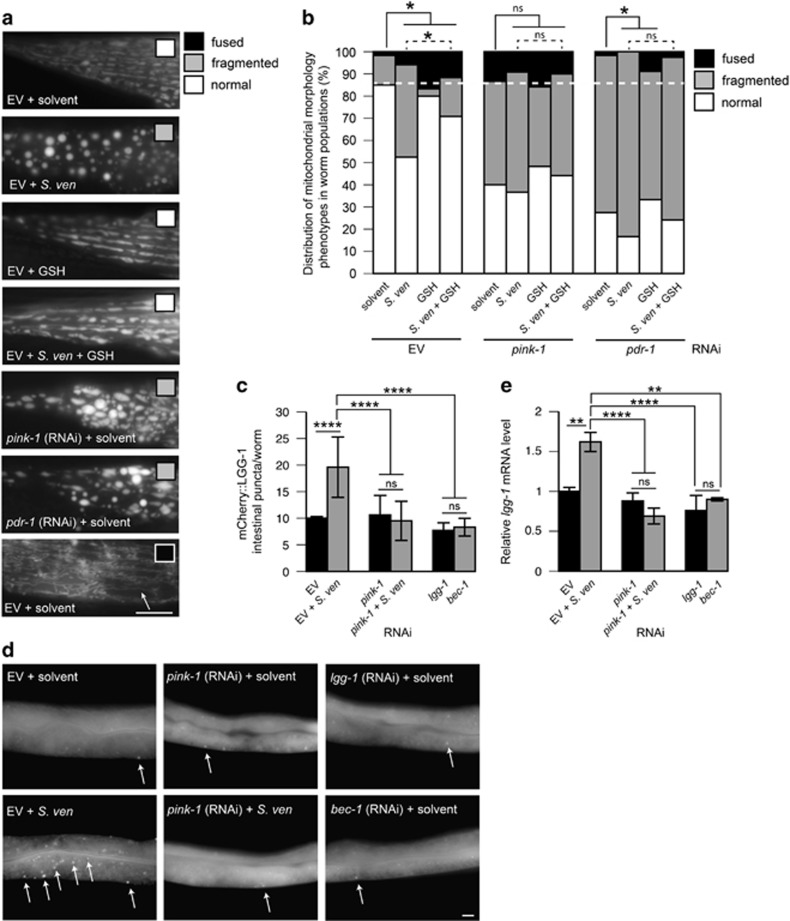
The *S. venezuelae* metabolite elicits GSH-tractable mitochondrial fragmentation and induces PINK-1-dependent autophagy. Nematodes were exposed to the bacterial metabolite chronically for all assays as described in the Figure 1 legend. RNAi was performed systemically using target genes as indicated in various experiments. (**a**) Animals expressing a mitochondrial-targeted GFP signal expressed in the *C. elegans* bodywall muscle cells were morphologically assessed during different combinations of *S. ven*, GSH, and RNAi treatment. Images depict representative phenotypes for each condition tested. The shaded boxes indicate the morphology represented in each [fused (also shown with an arrow), fragmented, or normal], as described in (**b**). Scale bar, 20 *μ*m. (**b**) The distribution of mitochondrial morphology, characterized as either normal (the majority of mitochondria are tubular), fragmented (the majority of mitochondria are circular or irregularly shaped), or fused (elongated or convoluted structures) for each condition tested is shown as a percentage in the population. The white-dashed line indicates the threshold for normal mitochondrial morphology in EV controls treated with solvent, EtAc. *n*=30 animals analyzed per treatment in 3–4 replicates. *P<0.05 was assessed by two-way ANOVA with Dunnet's *post hoc* test to compare different treatment groups with EtAc control within each RNAi treatment group. A separate (but not shown) statistical test using two-way ANOVA and Sidak's *post hoc* test reveals a statistical difference between solvent-treated *pink-1*(RNAi) and *pdr-1*(RNAi) when compared with the EV control in the context of mitochondrial fragmentation (**c** and **d**). Animals expressing an N-terminally fused mCherry::LGG-1 (LC3) construct were assessed for increased stress granule formation in the context of the *S. ven* metabolite and *pink-1*(RNAi); *lgg-1* and *bec-1* RNAi were used as negative controls. mCherry::LGG-1 puncta were counted in each of three rectangular boxes of 200 × 200 *μ*m; they were placed in tandem beginning with the most posterior region of the intestinal and extended toward the vulva. Arrows indicate representative puncta. *n*=30 animals analyzed per treatment in 3–4 replicates. ****P<0.001; one-way ANOVA followed by a Tukey's *post hoc* test. Data represented as mean±S.D. Scale bar, 10 *μ*m. (**e**) Animals exposed to empty vector (EV) or *pink-1*(RNAi) were assessed for *lgg-1* mRNA levels with or without *S. ven* metabolite treatment. This treatment occurred until the L4 stage. Three replicates comprises at least 100 animals each; 1 *μ*g of RNA was subjected to cDNA synthesis. Primers used for RT-qPCR are listed in Materials and methods. At least three stable reference genes were used. Data represented as mean±S.E.M. **P<0.01 and ****P<0.0001 were assessed using Q-base software

One signifier for altered autophagic capabilities is represented by mitochondrial morphology,^37, 39, 43^ which is normally tubular (Figures 5a and b).^44, 45^ We assessed this in response to metabolite exposure in animals bearing GFP fused to a mitochondrial import signal under the control of the muscle-specific *myo-3* promoter.^24^ Compared with solvent treatment, populations of control (EV) RNAi animals treated with metabolite have significantly greater fragmentation characterized by circularly shaped mitochondria, which is potentially indicative of increased mitophagic activity due to decreased mitochondrial fission and more rapid turnover of mitochondria (Figures 5a and b).^46, 47^ Only a few animals exhibited fused mitochondria (Figures 5a and b), which did not significantly change through metabolite exposure. When treated with GSH, metabolite-induced fragmentation was significantly reduced (Figures 5a and b), potentially linking GSH attenuation of metabolite-induced protein toxicity to mitochondrial morphology.

Next, we examined the regulation of mitochondrial morphology by PINK-1 and PDR-1. Compared with EV RNAi control populations, solvent-treated animals reduced for *pink-1* and *pdr-1* had significantly greater fragmentation (Figures 5a and b). These data support previously published research suggesting that declining loss of PINK-1 induced through RNAi can cause mitochondrial fragmentation.^39, 43, 45^ The proportion of animals exhibiting disordered or circular mitochondria in *pink-1*(RNAi) was not altered with metabolite treatment, but was significantly increased in *pdr-1*(RNAi) (Figures 5a and b), suggesting that reduction of *pdr-1* may sensitize mitochondria to toxic insults elicited through metabolite exposure, similar to *α*-synuclein-induced neurotoxicity (Figures 3a, d, and e). When RNAi-treated animals were supplemented with GSH, mitochondrial fragmentation was not modulated, suggesting that PINK-1 and PDR-1 are necessary for GSH activity in attenuation of metabolite-induced fragmentation.

The inability to attenuate metabolite-induced mitochondrial morphology by GSH in *pink-1*(RNAi) worms might be due to the epistatic nature of *pink-1* loss. Because PINK1 controls autophagy in other systems, increased fragmentation due to *pink-1* (RNAi) in *C. elegans* may represent an accumulation of fragmented mitochondria due to alterations in autophagic capabilities.^37, 42, 43, 48, 49^ To explore this further, we utilized transgenic nematodes expressing an autophagy reporter, mCherry::LGG-1 (homolog of LC3).^50^ mCherry::LGG-1 puncta formation in metabolite-treated animals was significantly higher in comparison with solvent only (EV) controls and *bec-1* and *lgg-1* RNAi (Figures 5c and d). This indicates that autophagy is induced in response to metabolite exposure and that observed mitochondria fragmentation may signify higher rates of mitochondrial turnover. Notably, animals with reduced *pink-1* no longer have increased mCherry::LGG-1 accumulation in the presence of the metabolite. When *lgg-1* was probed by qPCR to monitor transcriptional activity, a similar relationship was observed, suggesting that the induced autophagy observed in metabolite-treated animals is PINK-1 dependent (Figure 5e). Therefore, it is likely that the metabolite, perhaps through UPS perturbations, elicits an increase in autophagic activity and an increase in mitochondrial fragmentation due to higher mitochondrial turnover that is attenuated through GSH. This system is disrupted through *pink-1* loss-of-function wherein mitochondrial fragmentation occurs due to reduced capacity for stress-induced autophagy and potentially leads to protein damage and, in neuronal compartments, neurodegeneration.

## Discussion

Environmental contributors to neurodegenerative disease are less well studied than genetic potentiators. Nonetheless, characterization of environmental stressors offers the potential to enhance our understanding of idiopathic disease. Within bacteria, thousands of clades produce secondary metabolites, the majority of which are of unknown structure or activity and provide alluring targets for studies of unknown yet potentially ubiquitous stressors. Our work identified a potential microbial source for neurodegeneration represented in the *S. venezuelae* metabolite and implies that other bacteria may also produce neurodegeneration-inducing secondary metabolites.

Our studies suggest that ROS^25^ and UPS perturbations act upon the innate toxicity of disease-linked pathogenic proteins by observing degeneration in *C. elegans* neurons and a deleterious increase of phenotypic readouts in bodywall muscle cells expressing misfolded proteins. The presence of pathogenic proteins can be important in disease progression through alterations of threshold states^51, 52^ where homeostatic pathway dysregulation compounds over time. Consistent with this, no neurodegenerative phenotypes are observed in youthful animals in the absence of pathogenic protein expression, suggesting that the metabolite may act most strongly on threshold state animals. Mechanisms that link the UPS and ROS have yet to be fully explored, but our studies suggest that GSH regulation alters UPS activity and that this alteration is important for inducing toxic protein handling states. Furthermore, GSH appears to have a proactive role in compensating for UPS dysfunction, as GSH can protect against MG132 toxicity in the context of *α*-synuclein overexpression and dysregulation of GSH synthesis acts in manner similar to MG132 treatment. GSH attenuation is hypothesized to occur through repairing damaged cysteine residues,^32^ which occur as a result of metabolite exposure; shifting the GSH couple to a more reduced state might beneficially alter the neurodegenerative threshold state of *C. elegans* cells.^53^

Elucidating possible mechanisms for this metabolite might make use of pathways already defined for familial disease. It is a major hypothesis of disease pathogenesis that causative mutations within genes might be within very similar or even identical genetic pathways.^51^ Therefore, it is possible that UPS perturbations and ROS induction may collaborate with PINK1 and Parkin pathways in the context of Parkinsonism. In our studies, we defined a pathway comprises the UPS and GSH homeostasis that is epistatically regulated by loss of *pink-1*. The *C. elegans* Parkin homolog appears to act in parallel to PINK1 dysfunction, despite the importance of Parkin toward mitochondrial maintenance and in ameliorating *α*-synuclein stress. However, we hypothesize that due to the nature of *pdr-1* loss-of-function that seemingly parallel regulation of metabolite toxicity may actually be a synergistic toxic state where *pdr-1* loss-of-function interacts with *pink-1* loss-of-function in undefined, yet GSH-tractable, ways (as neurodegeneration in double mutants can be partially rescued by GSH).

Finally, we wished to provide a context by which *pink-1* loss-of-function may epistatically regulate toxicity associated with metabolite exposure by investigation of autophagic capabilities regulated by PINK-1. We discovered that the metabolite, as well as depletion of *pink-1* or *pdr-1*, induced mitochondrial fragmentation, which we hypothesized may be due to alterations in autophagic capabilities.^37, 42, 43, 48, 49^ However, although GSH attenuated fragmentation resulting from metabolite exposure, GSH did not attenuate metabolite-induced mitochondrial fragmentation in *pink-1*(RNAi) backgrounds. To explain this, we found that in *pink-1* (RNAi) backgrounds, LGG-1 recruitment and upregulation (which are induced in metabolite-treated animals) are impaired. This is consistent with evidence that decreased PINK1 signaling impairs stress-induced autophagy.^37^

These data as a whole indicate that the metabolite can induce proteostatic deficiencies through GSH-tractable UPS impairments, which may increase the need for bulk autophagy. This state produces fragmented mitochondria potentially as an indicator of mitochondria undergoing autophagy. The metabolite also elicits neurodegeneration in animals bearing pathogenic proteins and accumulation of damaged proteins in worm bodywall muscle cells. Reduction of PINK-1 signaling disrupts this paradigm and animals become intractable to GSH attenuation. We hypothesize that this reduction supersedes metabolite-induced neurotoxicity, potentially due to the nature of PINK-1 loss-of-function, loss of autophagy induction, and accumulation of mitochondrial fragmentation. In the future, research defining relationships among PINK-1, UPS, and autophagy in neurons may help to further elucidate the connection between these pathways and neurodegeneration. From a gene-by-environment perspective, it should be noted that three aspects of idiopathic PD: proteasome function deficiencies, depleted GSH intracellular concentrations, and mitochondrial dysfunction, mechanistically intersect within the literature^11, 20, 21, 32, 34^ and also when *S. venezuelae* metabolite disrupts cellular processes. Therefore, environmental stress may proceed via GSH-tractable intracellular deficiencies, which connect seemingly diverse homeostatic pathways.

## Materials and Methods

### *C. elegans* strains

*C. elegans* were grown and maintained using standard procedures.^54^ The following strains were provided by the CGC, which is funded by NIH Office of Research Infrastructure Programs (P40 OD010440): N2 (Bristol), DA1240 (*adIs1240*[P_*eat-4*_::GFP]), HA3 (*lin-15B*(*n765*); *nuIs11*[P_*osm-10*_::GFP, lin-15B(+)]), HA659 (*dpy-20*(*e1282*); *rtIs11*[P_*osm-10*_::GFP, P_*osm-10*_::HtnQ150, dpy-20(+)]), AM140 (*rmIs132* [P_*unc-54*_::Q35::YFP]), AM141 (*rmIs133* [P_*unc-54*_::Q40::YFP]), SD551 (*let-60(ga89)*), CB1402 (*unc-15(e1402)*), SJ4103 (*zcIs14*[P_*myo-3*_::GFP^MT^]), VC1024 (*pdr-1 (gk448*)), VK1093 (*vkEx1093*[P_*nhx-2*_::mCherry::LGG-1]), CL802 (*smg-1*(cc546); *rol-6(*su1006)), and CL4176 (*smg-1*(*cc546*); *dvIs27*[pAF29 (P_*myo-3*_::A*β*42, *rol-6*(su1006)]). Strains containing the alleles *pink-1(tm1779)* and *pdr-1(tm598)* were provided by the Mitani Lab through the National Bio-Resource Project, Japan. Strains BY250 (*vtIs7*[P_*dat-1*_::GFP]) and BY200 (*vtIs1*[P_*dat-1*_::GFP, rol-6(su1006)]) were kind gifts from Randy Blakely (Vanderbilt University). Other strains include UA44 (*baIn11*[P_*dat-1*_::*α*-synuclein, P_*dat-1*_::GFP]), UA96 (*unc-119(ed3)*; *baIn19* [P_*dat-1*_::CFP::CL-1; *unc-119*(+)]), UA198 (*baIn34* [P_*eat-4*_::A*β*_42,_P_*myo-2*_::mCherry]); *adIs1240*[P_*eat-4*_::GFP]), UA49 (*baIn2*[P_*unc-54*_:: *α*-synuclein::GFP, *rol-6*(*su1006*)]), UA196 (*sid-1*(*pk3321*); *baIn33*[P_*dat-1*_::*sid-1*, P_*myo-2*_::mCherry); *baln11*[P_*dat-1*_::GFP; P_dat-1_::*α*-synuclein]), UA226 (*pink-1*(*tm1779*); *vtIs1*[P_*dat-1*_::GFP, *rol-6*(*su1006*)]), UA227 (*pdr-1*(*tm598*); *vtIs1*[P_*dat-1*_::GFP, *rol-6*(*su1006*)]), UA86 (*pink-1*(*tm1779*); *baIn11*[P_*dat-1*_::*α*-synuclein, P_*dat-1*_::GFP]), UA88 (*pdr-1*(*tm598*); *baIn11*[P_*dat-1*_::*α*-synuclein, P_*dat-1*_::GFP]), UA271 (*pink-1*(*tm1779*); *pdr-1*(*tm598*); *baIn11*[P_*dat-1*_::*α*-synuclein, P_*dat-1*_::GFP]), UA279 (*pdr-1(gk448)*; *baIn11*[P_*dat-1*_::*α*-synuclein, P_*dat-1*_::GFP]), and UA280 (*pink-1(tm1779)*; *pdr-1(gk338); baIn11*[P_*dat-1*_::*α*-synuclein, P_*dat-1*_::GFP]).

### Construction of pink-1(tm1779), pdr-1 (tm598), and pdr-1(gk448) containing strains

When crossing mutant strains containing these alleles, the molecular lesions are easily probed using the following primers:

*pink-1(tm1779)* Forward: GTTACAAGGCGAGCCTGAAAG

*pink-1(*tm1779) Reverse: GAAGCCTCGGGCTTATTAAGG

*pdr-1(tm598)* Forward: CAGACAAATCATGCTTCTCCG

*pdr-1(tm598)* Reverse: CGTCTTCGCTCTGGCACACAC

*pdr-1(gk448)* Forward: CACTTACGCAAGTGCTTCTTCG

*pdr-1(gk448)* Reverse: GTACGTGAGTTAGAGCTGC

In all cases the mutant animals were crossed into strain UA44 (*baIn11* [P_dat-1_::*α*-synuclein, P_*dat-1*_::GFP]) to generate strains UA86, UA88, UA271, UA279, and UA280; and into BY200 (*vtIs1*[P_dat-1_::GFP, *rol-6*(su1006)]) to generate UA226 and UA227.

### Isolation and extraction of *S. venezuelae* metabolite

Metabolite was generated as previously described.^25^ Briefly, spores from the *S. venezuelae* strain (ARS NRRL ISP-5230) were inoculated in 5 liters of SYZ media in artificial seawater and grown at 30 °C in a shaker. Samples were harvested after 3 weeks. Cell debris was removed by centrifugation at 10 000 × *g* for 10 min and supernatants were sequentially passed through 4–6 PES filter membranes with the following range of pore sizes: 11, 6, 2.7, 1.7, 1.2, 0.7, 0.45, and 0.22 *μ*M. The resulting conditioned media was extracted with an equal volume of DCM using a separatory funnel (2–3 times). The DCM layer was collected, dried, and the residue was resuspended in EtAc to a 1000-fold concentrated stock solution compared with the original volume of conditioned media. We calculate working concentrations of metabolite of this concentrated stock solution. Therefore, a 1 × concentration is 1 *μ*l of metabolite resuspended in 1 ml EtAc. In our assays, chronic exposure utilizes a 5 × concentration for neurodegeneration assays and 20 × concentration for autophagy and mitochondrial morphology, while semi-acute soaking exposure associated with pathogenic protein aggregation utilizes 10–20 × concentrations.

### Chronic metabolite treatment

All drugs were provided at final concentrations as indicated in figures. Animals were either exposed to metabolite chronically or in a semi-acute manner (Supplementary Figure S1). The purpose of the chronic treatment paradigm, described here, was to provide consistent long-term exposure to the metabolite and was used for neurodegeneration associated with N2 (Bristol), UA96 (*unc-119(ed3)*; *baIn19* [P_dat-1_::CFP::CL-1; *unc-119*(+)]), BY250 (*vtIs7*[P_*dat-1*_::GFP]), UA44 (*baIn11*[P_*dat-1*_::*α*-synuclein, P_dat-1_::GFP]), UA196 (*sid-1*(*pk3321*); *baIn33*[P_*dat-1*_::*sid-1*, P_*myo-2*_::mCherry), DA1240 (*adIs1240*[P_*eat-4*_::GFP]), UA198 (*baIn34*[P_*eat-4*_::A*β*_42,_P_*myo-2*_::mCherry]), HA3 (*lin-15B*(*n765*); *nuIs11*[P_*osm-10*_::GFP, *lin-15B*(+)]), and HA659 (*dpy-20*(*e1282*); *rtIs11*[P_*osm-10*_::GFP, P_*osm-10*_::HtnQ150, *dpy-20*(+)]) as well as autophagy associated with the strain VC1093 (*vkEx1093*[P_*nhx-2*_::mCherry::LGG-1] and mitochondrial morphology associated with the strain SJ4103 (*zcIs14*[P_*myo-3*_::GFP^MT^]).

Metabolite solution [0.5 *μ*l (5 × ) or 2 *μ*l (20 × ) of the concentrated stock solution is reconstituted in 100 *μ*l EtAc] or EtAc (solvent control) alone was placed within the bacterial lawn and allowed to dry. After, mixed staged animals are placed on each treatment plate and allowed to reach gravid adulthood. These animals are used for egg laying. This is a pretreatment paradigm, which we have previously shown produces a stronger and more consistent result.^25^ Two days after hatching 100 *μ*l/4 ml agar of 5 × metabolite in the M9 salt buffer (3 mg/ml KH_2_PO_4_, 6 mg/ml Na_2_HPO_4_, 5 mg/ml NaCl, 1 mM MgSO_4_) or M9 (with appropriate amounts of EtAc) is supplemented to an NGM plate to refresh the metabolite without the need for transfer. Two days after this, synchronized adults are transferred to a new OP50-1 or HT115 RNAi bacteria plate with the appropriate treatment. This cycle continues every 2 days until analysis.

### Semi-acute metabolite treatment

All drugs were provided at final concentrations as indicated in figures. Animals were either exposed to metabolite chronically or in a semi-acute manner (Supplementary Figure S1). The purpose of the semi-acute treatment, described here, is to pulse the animals with a high dosage of metabolite over a short period of time by soaking animals in a metabolite solution followed by high-dosage chronic exposure and is associated with protein aggregation assays associated with the strains: N2 (Bristol), SD551 (*let-60(ga89)*), CB1402 (*unc-15(e1402)*), UA49 (*baIn2*[P_unc-54_:: *α*-synuclein::GFP, *rol-6*(*su1006*)]), AM140 (*rmIs132* [P_*unc-54*_::Q35::YFP]), AM141 (*rmIs133* [P_*unc-54*_::Q40::YFP]), UA4 (*baIn4*[P_*unc-54*_::Q82::GFP, *rol-6*(*su1006*)]), and CL4176 (*smg-1*(*cc546*) I; *dvIs27*[pAF29 (P_*myo-3*_::A*β*42, *rol-6*(su1006))]).

Young adults of any strain used for analysis were placed on bacterial lawns supplemented with metabolite in EtAc or EtAc alone. Before food runs scarce and at the point where the majority of animals are gravid, the contents of the plate were washed into a glass conical tube and hypochlorite treated for 5–10 min to dissolve adults but leave embryos unaffected. This solution was spun down in a table-top centrifuge at ~500 g. The supernatant was removed and washed with the M9 buffer 2–3 times; the final time the volume is brought down to less than 100 *μ*l. This volume is placed on a 4-ml NGM plate (35 mm) with approximately 10 *μ*l *E. coli* to provide a small amount of food overnight to prevent starvation. Twelve hours later, animals are washed from their plate with M9 into a glass conical tube to a final volume of 600 *μ*l. Added to this solution is 6–12 *μ*l of the 1000 × concentrated metabolite solution (controls are treated by adding 6 *μ*l of EtAc). Care must be taken to prevent the small amount of EtAc added to the solution from initially accumulating at the bottom of the tube (which will kill the animals) by gently shaking the glass conical tube with the cap off for ~1 min. Glass tubes are then placed at 20 °C on a gentle tube shaker for 8 h. After 8 h, the entire content of the tube is distributed to 4 ml NGM plates and allowed to dry. In all, 100 *μ*l *E. coli* bacteria is then placed on agar plates and allowed to dry. Worms are then placed in their respective incubators (20 °C for most or 16 °C for A*β*_42_ temperature-sensitive paralysis strains). Forty-eight hours later, metabolite in M9 is supplemented to the plates (36 h for A*β*_42_ temperature-sensitive paralysis strains or day 4 post hatching for PolyQ_35/40lines_).

For strains CL4176 (*smg-1*(*cc546*) I; *dvIs27*[pAF29 (P_*myo-3*_::A*β*42, *rol-6*(su1006))]) and CL802 (*smg-1*(cc546); *rol-6(*su1006)) conditioned media (see Isolation and extraction of *S. venezuelae* metabolite) is used instead of metabolite in EtAc because animals do not fare well in the presence of even mild amounts of EtAc. For these strains, SYZ growth media was used as a control.

### RNAi bacteria growth conditions and treatment

RNAi bacteria (HT115) containing the L4440 feeding vector are initially grown on LB tetracycline-ampicillin plates (10 mg/ml tryptone, 5 mg/ml yeast extract, 10 mg/ml NaCl, 15 mg/ml agar, 12.5 *μ*g/ml tetracycline, 100 *μ*g/ml ampicillin) for 16 h at 37 °C. Single colonies were grown in LB ampicillin broth (100 *μ*g/ml ampicillin) for 16 h at 37 °C. RNAi bacteria is then placed onto NGM IPTG-ampicillin analysis plates (NGM: 1 mM IPTG, 100 *μ*g/ml ampicillin) at 250 *μ*l/4 ml NGM for 24 h at 22 °C. The RNAi clones *pink-1(EED8.9), uba-1(C47E12.5), psmd-9(C44B7.1), rpn-2 (C23G10.4), rpt-1 (C52E4.4), rpt-4 (F23F1.8), pas-3 (Y110A7A.14), pbs-3 (Y38A8.2), lgg-1(C32D5.9), bec-1(T19E7.3)*, and L4440 (EV) were obtained from the Ahringer Library;^55^ *pdr-1(KO8E3.7)* was generated in our laboratory.^27^ In most cases, nematodes were exposed to RNAi bacteria for two generations. Proteasome RNAi analysis was postponed until the the L4 molt (~60 h post hatching) to exclude developmental impairments.

### Drug treatments

DiI (Biotium, Hayward, CA, USA) was used at a final concentration of 100 ng/*μ*l. MG132 (Cayman Chemical, Ann Arbor, MI, USA) was used at a concentration of 1–50 *μ*M (see Supplementary Figure S5A) in DMSO. BSO (Enzo Life Sciences, Ann Arbor, MI, USA) was utilized at a 1-mM concentration dissolved in water. Antioxidants were brought to a final concentration of 1 mM in NGM plates by dissolving in the media during preparation. These drugs were ascorbic acid (Sigma-Aldrich, St. Louis, MO, USA), uric acid (Alfa Aesar, Ward Hill, MA, USA), probucol (MP Biomedicals, Santa Ana, CA, USA), melatonin (Sigma-Aldrich), and GSH (Sigma-Aldrich). Probucol was dissolved in ethanol, the final volume of which did not exceed 1%. All GSH experiments were performed using drug plates with 1 mM concentration throughout the entirety of the experiment refreshed every 2 days.

### Analysis of dopaminergic neurodegeneration

This assay was performed on animals containing *α*-synuclein in dopaminergic neurons UA44 (*baIn11*[P_*dat-1*_::*α*-synuclein, P_*dat-1*_::GFP]), UA196 (*sid-1*(*pk3321*); *baIn33*[P_*dat-1*_::*sid-1*, P_*myo-2*_::mCherry); *baln11*[P_*dat-1*_::GFP; P_*dat-1*_::*α*-synuclein]), UA86 (*pink-1*(*tm1779*); *baIn11*[P_*dat-1*_::*α*-synuclein, P_dat-1_::GFP]), UA88 (*pdr-1*(*tm598*); *baIn11*[P_*dat-1*_::*α*-synuclein, P_*dat-1*_::GFP]), and UA271 (*pink-1*(*tm1779*);*pdr-1*(*tm598*); *baIn11*[P_*dat-1*_::*α*-synuclein, P_*dat-1*_::GFP]) or GFP alone animals BY250 (*vtIs7*[P_*dat-1*_::GFP]), BY200 (*vtIs1*[P_*dat-1*_::GFP, *rol-6*(su1006)]), UA226 (*pink-1*(*tm1779*); *vtIs1*[P_*dat-1*_::GFP, *rol-6*(*su1006*)]), UA227 (*pdr-1*(*tm598*); *vtIs1*[P_*dat-1*_::GFP, *rol-6*(*su1006*)]), UA279 (*pdr-1(gk448)*; *baIn11*[P_*dat-1*_::*α*-synuclein, P_*dat-1*_::GFP]), and UA280 (*pink-1(tm1779)*; *pdr-1(gk338); baIn11*[P_*dat-1*_::*α*-synuclein, P_*dat-1*_::GFP]). In all, 30–40 animals were assessed for the presence of six anterior soma as well as their associated processes. Animals with missing soma or missing processes are considered as degenerating. Data were assessed by two-way ANOVA using Tukey's *post hoc* test.

### Analysis of glutamatergic neurodegeneration

Animals containing the A*β*_42_ cleavage product expressed in glutamatergic neurons UA198 (*baIn34*[P_*eat-4*_::A*β*_42,_P_*myo-2*_::mCherry]); *adIs1240*[P_*eat-4*_::GFP]) or GFP alone DA1240 (*adIs1240*[P_*eat-4*_::GFP]). In all, 30–40 animals are assayed for the presence of five glutamatergic neurons in the tail as well as their associated canonical processes. Data were assessed by two-way ANOVA using Tukey's *post hoc* test.

### Analysis for lipophilic dye uptake

Huntingtin-induced neurodegeneration in the ASH-sensory neuron using the strains that contain the Huntingtin-Q_150_ repeat HA659 (*dpy-20*(*e1282*); *rtIs11*[P_*osm-10*_::GFP, P_*osm-10*_::HtnQ150, dpy-20(+)]) or GFP alone HA3 (*lin-15B*(*n765*); *nuIs11*[P_*osm-10*_::GFP, lin-15B(+)]) were assayed for colocalization between endogenous GFP in the ASH-sensory neuron with the red DiI lipophilic dye by soaking animals on the day of analysis for 1 h in a 1% EtOH, 100 ng/*μ*l DiI in M9 buffer at room temperature. In all, 30–40 animals were analyzed. Data were assessed by two-way ANOVA using Tukey's *post hoc* test.

### Analysis of *α*-synuclein aggregation

Thirty animals containing body wall *α*-synuclein UA49 (*baIn2*[P_*unc-54*_:: *α*-synuclein::GFP, *rol-6*(*su1006*)]) were assayed 60 h after semi-acute exposure to the metabolite. Aggregate numbers were qualitatively determined on a 0–3 scale (0 none, 3 many) in worm lines^35^ wherein the experimenter was blinded to the treatment condition. Data were assessed by Student's *t*-test.

### Analysis of PolyQ aggregation

Thirty animals containing PolyQ repeat length polymorphisms, AM140 (*rmIs132* [P_*unc-54*_::Q35::YFP]) and AM141 (*rmIs133* [P_*unc-54*_::Q40::YFP]), were scored for the actual number of YFP puncta aggregates present throughout the animal. Data were assessed by Student's *t*-test.

### Analysis for A*β*_42_ induced paralysis

Ninety CL4176 (*smg-1*(*cc546*) I; *dvIs27*[pAF29 (P_*myo-3*_::A*β*42, *rol-6*(su1006))]) or CL802 (*smg-1*(cc546); *rol-6(*su1006)) animals per replicate bearing temperature-sensitive repression of the A*β*_42_ cleavage product were upshifted to various temperatures equal to or greater than 23 °C, 36 h after semi-acute metabolite treatment (L3 larval stage). Twenty-four hours later, animals are monitored for loss of the ability to execute a full body rotation (this strain contains *rol-6*) every hour for 9–12 h while being gently prodded twice with a platinum wire at the head and the tail. Animals that fail to execute a fully body rotation are deemed paralyzed. Data were assessed by two-way ANOVA using Tukey's *post hoc* test (GraphPad Prism, La Jolla, CA, USA).

### Analysis of a UPS-targeted CFP in dopaminergic neurons

Twenty UA96 (*unc-119(ed3)*; *baIn19* [P_dat-1_::CFP::CL-1; unc-119(+)]) worms, which bear a dopaminergic neuron targeted CL-1 ‘degron' ubiquitination signal translationally fused to CFP, were used for a minimum of three replicate series per treatment group (chronic metabolite and/or pharmacological treatment) at day 7 post hatching. One PDE neuron from each animal was imaged using standardized magnification and exposure values across all animals in all treatment groups. A 70-pixel diameter circle (approximately 0.1 *μ*M per pixel) was placed such that it encompasses the nucleus and the majority of the cytosolic compartment of the neuron. Average fluorescence values across all pixels within the circle were measured. Pools of 20 nuclei per replicate were averaged. Statistical tests were performed using a two-way ANOVA with the mean and standard error of the mean of each treatment group.

### Fluorescence microscopy

Worms were immobilized with 3 mM levamisole and mounted on 2% agarose pads on a microscopic slide. Fluorescent microscopy was performed using a Nikon Eclipse E800 epifluorescence microscope equipped with an Endow GFP HYQ filter cube (Chroma Technology, Bellows Falls, VT, USA) or a Texas Red filter cube (Chroma Technology). A Cool Snap CCD camera (Photometrics, Tuscon, AZ, USA) driven by the MetaMorph software (Molecular Devices, Sunnyvale, CA, USA) was used to acquire images.

### Analysis of metastable protein allele *unc-15 (e1402)* and polyQ35 motility

Mixed populations of N2 worms and *unc-15(e1402)* animals or polyQ35 were maintained at 16 °C. Populations were synchronized by hypochlorite treatment. Twenty-four hours later animals were semi-acutely exposed to the metabolite solution in M9 buffer at the L1 stage for 8 h at 16 °C then placed upon a recovery plate until analysis at the young adult stage (generally 24 h after L4 molt). At the analysis date, animals were transferred from the recovery plate to a clean NGM plate to clear animals of bacteria for approximately 10 min. Approximately 10 clean animals were placed in the center of a scoring plate and measured for a minimum of 100 frames using the MBF Bioscience Wormlab System (Williston, VT, USA). Average motility (*μ*M/s) was measured for each worm. Worms that did not move consistently or changed directions too frequently (less than 10 *μ*M/second) were excluded from the analysis. In all, 40–50 animals were analyzed per each treatment in total. Two-way ANOVA statistical test was performed with a Tukey's *post hoc* test (paramyosin) or a Student's *t*-test (polyQ35) was performed to compare with and without metabolite groups.

### Analysis of brood size and development in the metastable allele *let-60(ga89)*

*let-60(ga89)* animals have both reduced brood sizes and developmental delay. To measure both we maintained either *let-60(ga89)* animals or N2 animals at 16 °C at mixed populations. We hypochlorite synchronized animals. Twenty-four hours later we performed a semi-acute exposure. Worms were maintained at 16 °C throughout the entire experiment. At the young adult stage, we then placed a solitary worm in a 24-well plate for 24 h to lay eggs. After 24 h, the adult worm was cleared from the plate. Plates were then supplemented every 24 h with fresh metabolite solution. Ninety-six hours later, the number of animals per brood that had attained adulthood were counted. Each replicate contains 24 worm broods that were averaged. Each treatment group was normalized to the N2 solvent control. At least three replicates were used. Statistics on the mean and standard error of the mean were performed using a two-way ANOVA with a Tukey's *post hoc* test.

### Mitochondrial morphology analysis

SJ4103 (*zcIs14*[P_*myo-3*_::GFP^MT^]) animals were exposed for two generations of RNAi treatment (EV, *pink-1* or *pdr-1*) in the presence of the metabolite or solvent control. Animals were treated (Supplementary Figure S1B) for 7 days post hatching using a 20 × concentration of metabolite by chronic supplementation to the worm media. For GSH treatment, animals were placed on plates with 1 mM GSH dissolved within the media for the entirety of the analysis. Three muscle cells were analyzed per worm and thirty worms were analyzed/experiment. Data were analyzed by two-way ANOVA and Dunnett's and Sidak's *post hoc* test. Normal cells have ordered, ribbon-shaped mitochondria with few circular forms. Fragmented cells consist of disorganized circular forms and fused mitochondria have many ribbon-shaped mitochondria connected in a long labyrinthine formation.^44^

### Autophagy analysis

For autophagy analysis, 30 VK1093 (P_*nhx-2*_::mCherry::LGG-1) transgenic animals were analyzed at day 4 post hatching following 20 × metabolite treatment. To count puncta of mCherry::LGG-1, three 200 × 200 *μ*m boxes were assigned from posterior to anterior along the intestine. The first box was placed at region at the most posterior portion of the intestine. The second was placed anterior to this position, extending toward the vulva, etc. Data were analyzed by one-way ANOVA and Tukey's *post hoc*.

### RT-qPCR and RNA extraction

RT-qPCR was performed in accordance with MIQE standards. mRNA from at least 100 L4 or young adult N2 animals exposed to RNAi conditions was collected after washing worms of RNAi bacteria in water at least twice. RNA extraction was performed using protocols for either Tri Reagent with DNAse treatment^33^ or RNAzol (similar extraction methods were used within each experiment) (Molecular Research Center). In all, 1 *μ*g of RNA was used for cDNA synthesis using the iScript RT supermix for RT-qPCR (Bio-Rad, Hercules, CA, USA). Each primer pair used to probe *pink-1, pdr-1*, or *lgg-1* as well as reference genes for *cdc-42, pmp-3, tba-*1, and *snb-*1 was confirmed for at least 90–110% efficiency in a standard curve on N2 cDNA. RT-qPCR was performed with IQ-SYBR Green Supermix (Bio-Rad) with the CFX96 RealTime System (Bio-Rad). Three technical replicates and three independent biological replicates were utilized in these studies. Statistics were calculated using qBasePLUS version 2.6 (Biogazelle, Gent, Belgium). Primers used for these assays are as follows:

*pink-1* Forward: GCTCTACGAATTGCTCGCCTTG

*pink-1* Reverse: CAATTCTATTCGGCCATTCAGTGC

*pdr-1* Forward: ACACCTGCAACACAAATCATGC

*pdr-1* Reverse: GACTAGAACAGAGGTTGACGAGC

*lgg-1* Forward: CCACAAACCATGACCACAATG

*lgg-1* Reverse: CGACCTCTCCTCCATACA

*snb-1* Forward: CCGGATAAGACCATCTTGACG

*snb-1* Reverse: GACGACTTCATCAACCTGAGC

*cdc-42* Forward: CTGCTGGACAGGAAGATTACG

*cdc-42* Reverse: CTCGGACATTCTCGAATGAAG

*pmp-3* Forward: GTTCCCGTGTTCATCACTCAT

*pmp-3* Reverse: ACACCGTCGAGAAGCTGTAGA

*tba-1* Forward: GTACACTCCACTGCTCTGCTGACAAG

*tba-1* Reverse: CTCTGTACAAGAGGCAAACAGCCATG

### Statistical analyses

Statistical tests were performed using GraphPad Prism (San Diego, CA, USA) software.

## Abbreviations

*α*-syn: *α*-synuclein
A*β*: amyloid beta
AA: ascorbic acid
BSO: butathionine sulfoximine
DCM: dichloromethane
EtAc: ethyl acetate
EV: empty vector
GFP: green fluorescent protein
GSH: glutathione
MT: melatonin
Pro: probucol
RNAi: RNA interference
ROS: reactive oxygen species
*S. ven*: *Streptomyces venezuelae*
UAc: uric acid
UPR: unfolded protein response
UPS: ubiquitin proteasome system
YFP: yellow fluorescent protein
