# Supplementary material for: A bacterial metabolite induces glutathione-tractable proteostatic damage, proteasomal disturbances, and PINK1-dependent autophagy in C. elegans
Source: Cell Death Dis. 2015 Oct 15;6(10):e1908–. doi: 10.1038/cddis.2015.270 (PMC4632299; doi:10.1038/cddis.2015.270)
Supplement: Supplementary Figures [file cddis2015270x1.pdf]

Supplementary information

Five supplemental figures are included within this single document:

Figure S1.

Figure S2.

Figure S3.

Figure S4.

Figure S5.

Figure S1.

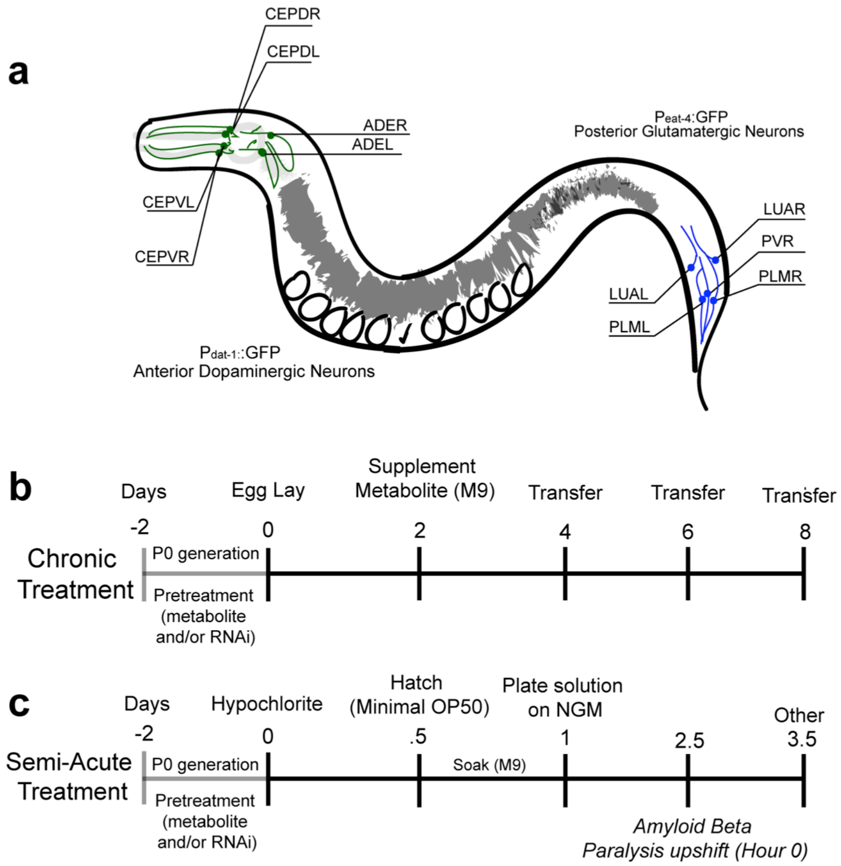

Figure S1. Worm morphology and *S. venezuelae* treatment paradigms. a. Depiction of *C. elegans* anterior dopaminergic neurons and posterior glutamatergic neurons assessed for neurodegeneration. **b.** Chronic treatment of animals to the *S. ven* metabolite is described in the Supplementary Material and Methods. Briefly, the P0 generation of animals was treated with either RNAi and/or *S. ven* metabolite prior to egg laying on day 0. This pre-treatment paradigm with either RNAi or metabolite produces a stronger effect in the F1 generation. Metabolite was supplemented every 2 days (the first supplementation is in M9 salt buffer directly to the plate, while the proceeding supplementations are added to the *E. coli* OP50 lawn and dried prior to animal transfer). **c.** Semi-acute treatment of animals with the *S. ven* metabolite is described in the Supplementary Methods. Briefly, the P0 generation of animals was treated with either RNAi and/or *S. ven* metabolite prior to hypochlorite bleach synchronization on day 0. Synchronized eggs were placed on a new 35 mm NGM plate seeded with ~10μL OP50 for 12 hours until hatching. After 12 hours, animals were soaked in a 10-15X solution of the metabolite in M9 or unextracted conditioned *S. ven* media (Aβ paralysis) for 8 hours. Equal volume of solution was partitioned to 3-4 plates per treatment group and analyzed at various time points.

Figure S2.

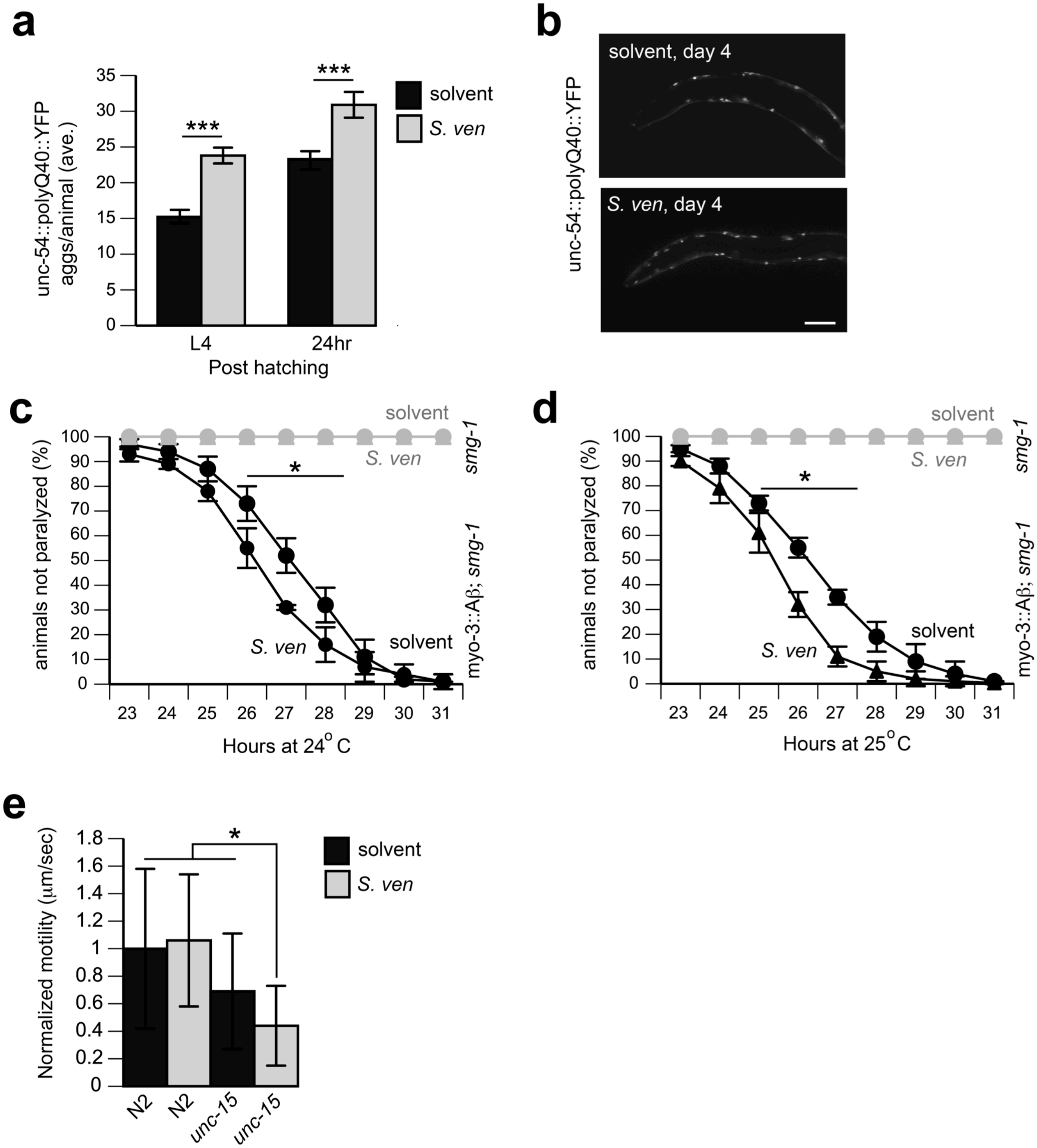

Figure S2. The *S. venezuelae* metabolite induces proteostasis disruption. Nematodes were treated acutely (as described in Figure S1c) for these assays. **a.** Animals expressing a polyglutamine-40 tract (Q_{40}) conjugated to YFP within the *C. elegans* bodywall muscle cells under the control of the $P_{unc\text{-}54}$ promoter were assayed at time points after treatment corresponding to the L4 larval stage and 24-hours after L4 by counting the number of aggregates present per animal. Data represented as mean ± S.E.M.; n=30 animals per treatment assessed in 3-4 replicates. **P<0.01. Data were assessed by Student's *t*-test. **b.**

Representative *C. elegans* bodywall muscle cells expressing polyQ$_{40}$::GFP treated with solvent or *S. ven* metabolite. Scale bar, 100 µm. **c, d.** Animals expressing Aβ_{42} peptide under the control of the bodywall muscle promoter (P$_{myo\text{-}3}$) using a temperature-repression system (*smg-1*) were upshifted to 24 and 25 °C at the L3 larval stage to induce expression of Aβ. In parallel, animals that do not express Aβ were treated identically (overlapping grey lines) but did not exhibit paralysis. Treatment was with *S. ven* metabolite or solvent. n=90-120 animals per treatment, replicated 3-4 times. Data represented as mean ± S.E.M. assessed using Two-Way ANOVA and Tukey's *post hoc* test to assess for significance between each time point in the analysis among every other time point. *P<0.05 for time points indicated. **e.** Animals bearing the metastable *unc-15(e1402)* allele or N2 (Bristol) animals were exposed to the metabolite or solvent control and tracked with the MBF Bioscience Wormlab System for motility (µm/second) on a clean agar plate. n=40-50 animals. Data was normalized to N2 solvent control animals and represented as mean ± S.D. was assessed by Two-Way ANOVA with Tukey's *post hoc* test; *P<0.05.

Figure S3.

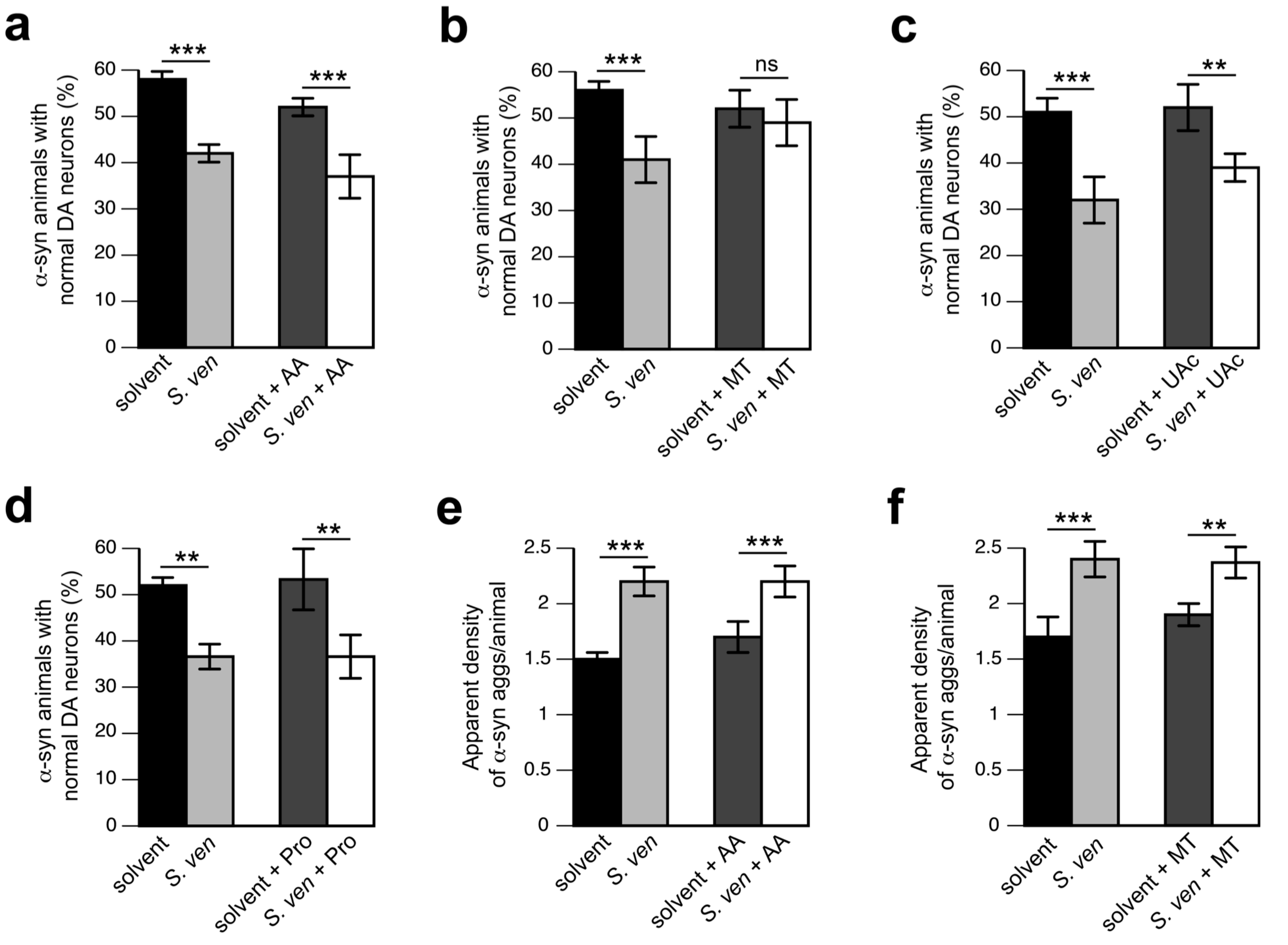

Figure S3. Various antioxidants have negligible remitting effects on proteostasis disruption in the context of the *S. venezuelae* metabolite. Anti-oxidants were dissolved in the worm NGM agar to a final concentration of 1mM for all experiments conducted. These anti-oxidants are ascorbic acid (AA), melatonin (MT), uric acid (UAc) and probucol (Pro). Probucol was first dissolved in ethanol and plates were brought to a final volume of ethanol not exceeding 1%. **a, b, c, d.** Animals expressing α-syn in the dopaminergic neurons were treated with the *S. ven* metabolite, as described in Figure 1a, were assessed for neurodegeneration in the context of 1mM AA, 1mM MT, 1mM UAc and 1mM Pro. Data represented as mean ± S.D.; n=30 animals analyzed per treatment in 3-4 replicates. $^{**}P<0.01$, $^{***}P<0.001$ was assessed by Two-Way ANOVA with Tukey's *post hoc* test. **e, f.** Animals expressing α-syn::GFP in the bodywall muscle cells were treated in a similar manner to the *S. ven* metabolite as described in Figure 2a were assessed for apparent aggregate density in the presence of 1mM AA or 1mM MT. Data are represented as the mean ± S.D.; n=30 animals analyzed per treatment in 3-4 replicates. $^{**}P<0.01$, $^{***}P<0.001$ was assessed by Two-Way ANOVA with Tukey's *post hoc* test.

Figure S4.

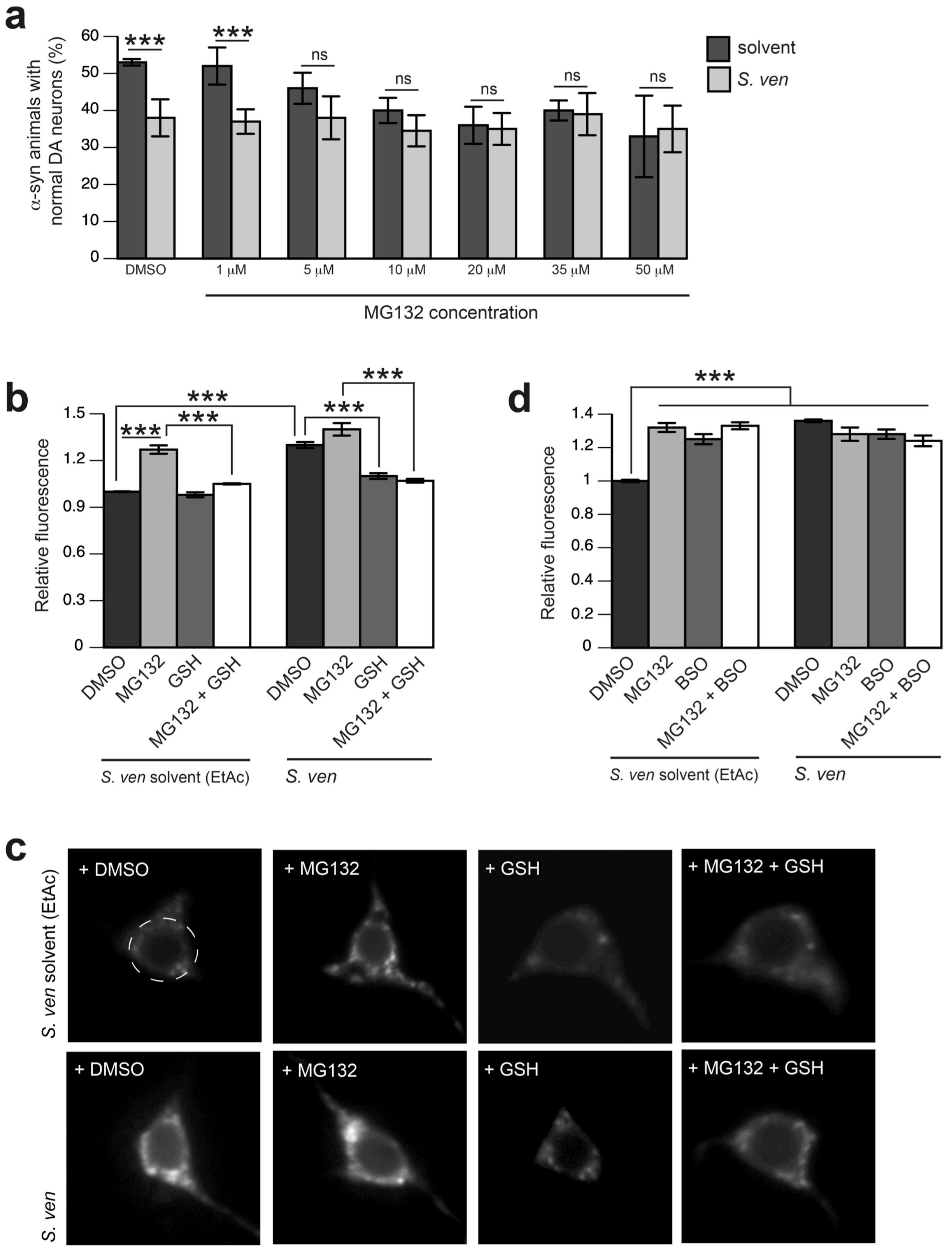
a
α-syn animals with normal DA neurons (%)
60
50
40
30
20
10
0
***
***
ns
ns
ns
ns
ns
solvent
S. ven
DMSO
1 μM
5 μM
10 μM
20 μM
35 μM
50 μM
MG132 concentration
b
Relative fluorescence
1.5
1.2
0.9
0.6
0.3
0
***
DMSO
MG132
GSH
MG132 + GSH
S. ven solvent (EtAc)
S. ven
d
1.4
1.2
1
0.8
0.6
0.4
0.2
0
BSO
MG132 + BSO
c
+ DMSO
+ MG132
+ GSH
+ MG132 + GSH

Figure S4. GSH attenuates MG132 and metabolite-induced proteasomal dysfunction in *C. elegans* PDE neurons. a. Animals were treated with 1 - 50 μM MG132 (with .1% DMSO serving as a solvent control) in conjunction with metabolite exposure. Animals were placed on MG132 concentrations at the larval L4 stage to exclude the possibility of developmental defects. Data represented as mean ± S.D; n=30 animals analyzed per treatment in 3-4 replicates. *** P<0.001 was assessed by Two-Way ANOVA with Tukey's *post hoc* test. **b, c.** Animals with a fluorescent CFP molecule conjugated to a degradation (CL-1) signal expressed in dopaminergic neurons under the *dat-1* promoter were treated to combinations of 10 μM MG132, metabolite and/or 1mM GSH. Representative images are shown in **c**. A 70-pixel diameter circle (approximately .1 μM per pixel) was placed around each nucleus and the majority of the surrounding cytosol. This quantitative circle area is a one-size-fits-most estimation for fluorescence (as shown in the upper left panel). Pixels within the circle were calculated into an average fluorescence value. n=20 neurons per replicate normalized to solvent only control. 3-4 replicates were performed. Data represents mean ± S.E.M. *** P<0.001 was assessed by Two-Way ANOVA with Tukey's *post hoc* test. **d.** Animals with a fluorescent CFP molecule conjugated to a degradation (CL-1) signal expressed in dopaminergic neurons under the *dat-1* promoter were treated to combinations of 10 μM MG132, metabolite and/or 1mM BSO. n=20 neurons per replicate normalized to solvent only control. 3-4 replicates were performed. Data represents mean ± S.E.M. *** P<0.001 was assessed by Two-Way ANOVA with Tukey's *post hoc* test.

Figure S5.

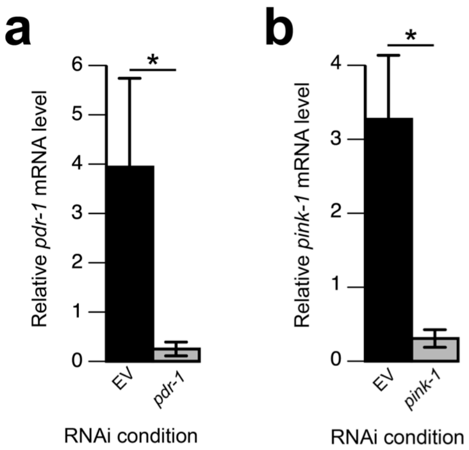

Figure S5. Confirmation of knockdown of *pdr-1* and *pink-1* as measured by RT-qPCR. a, b. N2 animals were exposed to *pdr-1*(RNAi) or *pink-1*(RNAi) subjected to RT-qPCR against *pdr-1* and *pink-1* to confirm adequate expression reduction. Primers used are listed in the Methods Section. Methods for RNA extraction and cDNA synthesis are described in the Methods Section as well. At least three replicates comprised of 1μg of RNA were tested against 2-3 reference genes. Data represented as mean ± S.E.M *P<0.05 was assessed using Q-base software.
